# Nonlinear Inertia Weighted Teaching-Learning-Based Optimization for Solving Global Optimization Problem

**DOI:** 10.1155/2015/292576

**Published:** 2015-09-02

**Authors:** Zong-Sheng Wu, Wei-Ping Fu, Ru Xue

**Affiliations:** ^1^School of Mechanical and Precision Instrumental Engineering, Xi'an University of Technology, Xi'an, Shaanxi 710048, China; ^2^School of Information Engineering, Tibet University for Nationalities, Xianyang, Shaanxi 712082, China

## Abstract

Teaching-learning-based optimization (TLBO) algorithm is proposed in recent years that simulates the teaching-learning phenomenon of a classroom to effectively solve global optimization of multidimensional, linear, and nonlinear problems over continuous spaces. In this paper, an improved teaching-learning-based optimization algorithm is presented, which is called nonlinear inertia weighted teaching-learning-based optimization (NIWTLBO) algorithm. This algorithm introduces a nonlinear inertia weighted factor into the basic TLBO to control the memory rate of learners and uses a dynamic inertia weighted factor to replace the original random number in teacher phase and learner phase. The proposed algorithm is tested on a number of benchmark functions, and its performance comparisons are provided against the basic TLBO and some other well-known optimization algorithms. The experiment results show that the proposed algorithm has a faster convergence rate and better performance than the basic TLBO and some other algorithms as well.

## 1. Introduction

Most of the swarm intelligent optimization studies and applications have been focused on nature-inspired algorithms. Numerous population-based and nature-inspired optimization algorithms have been presented, such as the Ant Colony Optimization (ACO), Genetic Algorithm (GA), Particle Swarm Optimization (PSO), Artificial Bee Colony (ABC), and Differential Evolution (DE). These optimization algorithms are based on different natural phenomena. ACO works based on the behavior of ant colony searching foods from the source to a destination [1, 2]. GA applies the theory of Darwin based on the survival of the fittest to the optimization problems [3, 4]. PSO emulates the collaborative behavior of birds flocking and fish schooling in searching for foods [5–7]. ABC uses the foraging behavior of a honey bee [8–10]. DE derived from the Genetic Algorithm, which is an efficient global optimizer in the continuous search domain [11, 12]. These algorithms have been applied to many engineering optimization problems and proven effective in solving specific types of problems. However, various algorithms have their own advantages and disadvantages in solving diverse problems. Generally, a good optimization algorithm should possess the three essential conditions. First, the algorithm has the ability of obtaining the true global optima value. Second, the convergence speed of the algorithms should be fast. Third, the program should have a minimum of control parameters so that it will be easy to use. If an optimization algorithm meets the above three conditions at the same time, it would be a great algorithm. Some optimization techniques often achieve global optima results but at the cost of the convergence speed. Those algorithms tend to focus on the quality of computational results rather than the convergence speed. However, the higher calculation accuracy and faster convergence speed are the ultimate aim in the practical applications.

Recently, Rao et al. [13, 14] proposed a teaching-learning-based optimization (TLBO) algorithm, inspired by the phenomenon of teaching and learning in a class. The TLBO requires only the common control parameters like population size and numbers of generation and that does not require any algorithm-specific control parameters; that is, it is a parameter-less algorithm [15]. Thus, there is no burden of tuning control parameters in the TLBO algorithm. Hence, the TLBO algorithm is simpler and more effective and involves relatively less computational cost. What it is more important is that the TLBO algorithm has the ability to achieve better results at comparatively faster convergence speed to other algorithms mentioned above. Therefore, the TLBO algorithm has been successfully applied in diverse optimization fields such as mechanical engineering, task scheduling, production planning and control, and vehicle-routing problems in transportation [16–20]. Similar to other swarm intelligent optimization algorithms, the basic TLBO can be improved further and further. In order to improve the performance of TLBO, several variants of the TLBO have been proposed. Rao and Patel presented an elitist TLBO (ETLBO) algorithm [15] to solve complex constrained optimization problems and used a modified version of TLBO algorithm [17] to solve the multiobjective optimization problem of heat exchangers. Sultana and Roy [19] proposed a quasioppositional teaching-learning-based optimization (QOTLBO) methodology in order to find the optimal location of the distributed generator to simultaneously optimize power loss, voltage stability index, and voltage deviation of radial distribution network. Ghasemi et al. [20] used Lévy mutation strategy based on TLBO for optimal settings of optimal power flow problem control variables. Furthermore, some improved TLBO algorithms have been proposed to solve the global function optimization problem [21–24] and the multiobjective optimization problem [17, 25, 26].

In this paper, we propose a novel improved TLBO, which is called nonlinear inertia weighted TLBO (NIWTLBO). A nonlinear inertia weighted factor is introduced into the basic TLBO to control the memory rate of learners, and another dynamic inertia weighted factor is used to replace the original random number in teacher phase and learner phase. So, as a result, the NIWTLBO has faster convergence speed and higher calculation accuracy for most of these optimization problems than the basic TLBO. The performance of NIWTLBO for solving global function optimization problems is compared with basic TLBO and other optimization algorithms. The analysis results show that the proposed algorithm outperforms most of the other algorithms investigated in this paper.

The rest of this paper is organized as follows.  Section 2 describes the basic TLBO algorithm in detail. In Section 3, the proposed NIWTLBO algorithm will be introduced. And Section 4 provides numerical experiments and results demonstrating the performance of NIWTLBO in comparison with other optimization algorithms. Finally, our conclusions are mentioned in Section 5.

## 2. Teaching-Learning-Based Optimization

The basic TLBO algorithm mainly consists of two parts, namely, the teacher phase and the learner phase. In teacher phase, the students can learn from the teacher to make their knowledge level closer to the teacher's. In learner phase, the students can learn from the interaction of other individuals to increase their knowledge. In the TLBO algorithm, a group of learners is considered as a population. Each learner is analogous to an individual of the evolutionary algorithm. The different subjects offered to the learners are considered as design variables of the optimization problem. A learner's result is analogous to the fitness value of the objective function for optimization problems. The best learner (i.e., the best solution in the entire population) is considered as the teacher. The best solution is the best value of the objective function of the given optimization problem. The design variables are the input parameters of the objective function.

The process of basic TLBO algorithm is described below.

### 2.1. Initialization

The notations used in TLBO are described as follows:

 
*NP* is number of learners in a class (i.e., population size).
 
*D* is number of subjects offered to the learners (i.e., dimensions of design variables).
 MAXITER is maximum number of allowable iterations.
 
*X*_*i*,*k*_ = (*X*_*i*,*k*,1_, *X*_*i*,*k*,2_,…, *X*_*i*,*k*,*j*_,…, *X*_*i*,*k*,*D*_) denotes a learner in class (i.e., the individual in the population) at any iterator *i*.
 
*X*_*i*,*k*,*j*_ denotes the result of *j*th subject offered to *k*th learner at *i*th iterator. *X*_*i*,teacher_ represents the teacher, that is, the best learner in a class at *i*th iterator.

The population *X* is randomly initialized by a search space bounded by *NP* × *D* matrix. The values of *X*_*i*,*k*,*j*_ are assigned randomly using the equation

(1) $$\begin{array}{c} X_{0,k,j}=L_{j}+rand\times \left(U_{j}-L_{j}\right), \end{array}$$

where *k* = 1,2, 3,…, *NP* and *j* = 1,2, 3,…, *D*. The rand represents a uniformly distributed random variable within the range [0,1]. *L*_*j*_ ∈ (*L*_1_, *L*_2_, *L*_3_,…, *L*_*D*_) represents the lower bound of design variable. *U*_*j*_ ∈ (*U*_1_, *U*_2_, *U*_3_,…, *U*_*D*_) represents the upper bound of design variable.

### 2.2. Teacher Phase

In this phase, the algorithm simulates the students learning from teachers. A good teacher can bring his or her learners up to his or her level in terms of knowledge. Hence, the mean result of a class may increase from a low level to the teacher's level. But, in fact, it is impossible that the mean result of a class reaches the teacher's level. Because of the individual differences and the forgetfulness of memory, the learners cannot gain all the knowledge of the teacher. A teacher can increase the mean result of a class to a certain value which depends on the capability of the whole class.

Let *M*_*i*,*j*_ = (1/*NP*)(∑_*k*=1_^*k*=*NP*^ *X*_*i*,*k*,*j*_) be the mean result of the learners on a particular subject “*j*” (*j* = 1,2,…, *D*) and let *X*_*i*,teacher_ be the teacher at any iteration *i*. *X*_*i*,teacher_ will try to move mean *M*_*i*,*j*_ towards its own level which is the new mean. Difference_Mean_*i*,*k*,*j*_ is the difference between the existing mean result of each subject and the corresponding result of the teacher for each subject at the iteration *i*. The solution is updated according to the difference between the existing and the new means given by

(2) $$\begin{array}{c} Difference\_Mean_{i,k,j}=r_{i}\left(X_{i,teacher,j}-T_{F}M_{i,j}\right), \end{array}$$

(3) $$\begin{array}{c} T_{F}=round\left[1+\mathrm{rand}\left(0,1\right)\left\{1-2\right\}\right], \end{array}$$

(4) $$\begin{array}{c} X_{i,k,j}^{new}=X_{i,k,j}^{old}+Difference\_Mean_{i,k,j}, \end{array}$$

where *X*_*i*,teacher,*j*_ is the result of the teacher in subject *j* at the iteration *i*. *r*_*i*_ is a random number in the range [0, 1], and *T*_*F*_ is the teaching factor, which decides the value of mean to be changed. *T*_*F*_ can be either 1 or 2. The values of *r*_*i*_ and *T*_*F*_ are generated randomly in the algorithm and both of these parameters are not supplied as input to the algorithm.

In every iteration, *X*_*i*,*k*,*j*_^new^ is the updated value of *X*_*i*,*k*,*j*_^old^. Because the optimization problem is a minimization problem, our goal is to find the minimum of *f*. If the new value gives a better function value, then the old value is updated with the new value. The updated formula is given as

(5) iffXi,total_knew<fXi,total_koldXi,k,jold=Xi,k,jnewend  if,

where *X*_*i*,total_*k*_^new^ and *X*_*i*,total_*k*_^old^ represent the new and old total result of *k*th student at the iteration *i*, respectively. All the accepted new values at the end of the teacher phase become the input to the learner phase.

### 2.3. Learner Phase

In learner phase, the algorithm simulates the learning of the learners through interaction among themselves. A learner interacts randomly with other learners to increase his or her knowledge. If a learner has more knowledge than others, the other learners can quickly achieve new knowledge by learning from him or her to increase their level. In this learning process, two learners are randomly selected. One is *X*_*i*,*k*_ and another is *X*_*i*,*q*_, *k* ≠ *q*. The updated formula is given as

(6) $$\begin{array}{c} X_{i,k,j}^{new}=\left\{\begin{array}{cc} X_{i,k,j}^{old}+r_{i}\left(X_{i,k,j}-X_{i,q,j}\right) & if\;\;f\left(X_{i,total\_k}\right)<f\left(X_{i,total\_q}\right),\\ X_{i,k,j}^{old}+r_{i}\left(X_{i,q,j}-X_{i,k,j}\right) & otherwise, \end{array}\right. \end{array}$$

where *r*_*i*_ is a random number in the range [0, 1]. *X*_*i*,total_*k*_ and *X*_*i*,total_*q*_ represent the total result of *k*th student and *q*th student at the iteration *i*, respectively. Accept the new value if it improves the value of the objective function. Similarly, use (5) to update the learner.

In each iteration of the TLBO, it is necessary to detect the repeated solution to the entire population. If there is a repeated solution, it needs to remove the repeated solution and generate a new individual randomly. Hence, it will expand the diversity of populations and avoid premature convergence of the algorithm. After a number of generations, the knowledge level of the entire class is smoothly approximated to a point that is considered the teacher, and the algorithm converges to a solution.

### 2.4. Algorithm Termination

The algorithm is terminated after MAXITER iterations. The details of TLBO algorithm can be referred to in literature [13, 14].

## 3. Nonlinear Inertia Weighted Teaching-Learning-Based Optimization

The basic TLBO algorithm is based on teaching-learning phenomenon of a classroom. In the teacher phase, the teacher tries to shift the mean of the learners towards himself or herself by teaching. In the learner phase, learners improve their knowledge by interaction among themselves. In the process of the teaching-learning, learners improve their level by accumulating knowledge. In other words, they learn new knowledge based on existing knowledge. In the real world, the teacher tends to wish that his or her students should achieve the knowledge equal to him in fast possible time. But it is impossible for a student because of his or her forgetting characteristics. In fact, a student usually forgets a part of existing knowledge due to the physiological phenomena of the brain. With increasing the iteration numbers of learning, more and more existing knowledge will be remembered. As the learning curve presented by Ebbinghaus, it describes how fast learning knowledge is in learning process. The sharpest increase occurs after the first try and then gradually evens out, meaning that less and less new knowledge is retained after each repetition. Like the forgetting curve, the learning curve is exponential. So it is necessary to add a memory weight to the existing knowledge of the student for simulating this learning scenario. According to this phenomenon, a nonlinear inertia weighted factor *w* is introduced into (4) and (6) in the basic TLBO, and this factor is considered as memory weighted factor which controls the memory rate of learners. This nonlinear inertia weighted factor will scale the existing knowledge of the learner for computing the new value. In contrast to the basic TLBO, in our algorithm the part of previous value of the learner is decided by a weighted factor *w* while computing the new learner value.

**Figure 1:**
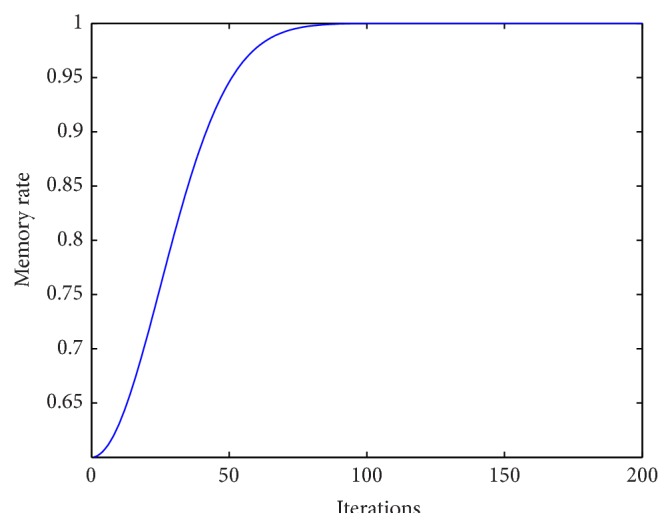
The memory rate curve.

Accordingly, to meet the characteristic of memory to conform to the learning curve, the* nonlinear inertia weighted factor w* (i.e., memory rate) is nonlinearly increased from *w*_min_ to 1.0 over time, whose value is given as

(7) $$\begin{array}{c} w=1-\exp\left(\frac{-iter^{2}}{2\times {\left(MAXITER/8\right)}^{2}}\right)\left(1-w_{min}\right), \end{array}$$

where iter is the current iteration number, MAXITER is the maximum number of allowable iterations, and *w*_min_ ∈ [0.5,1] is the minimum value of* nonlinear inertia weighted factor w*. The value *w*_min_ should be above 0.5 (here it is selected 0.6), or the individuals are worse due to remembering too little existing knowledge at first. Hence, if the value *w*_min_ is too small, the algorithm could not converge to the true global optimal solution. *w* curve (i.e., memory rate curve) is shown as Figure 1. The* nonlinear inertia weighted factor w* is applied to the new equations shown as (10) and (11). In this modified TLBO, the individuals try to sample diverse zones of the search space during the early stages of the search. During the later stages, the individuals adjust the movements of trial solutions finely so that they can explore the interior of a relative small space.

In the teacher phase, in order to obtain a new set of better learners, the difference between the existing mean result and the corresponding result of the teacher is added to the existing population of learners. Similarly, to obtain a new set of better learners in the learner phase, two learners are selected randomly, and the difference between their result of each corresponding subject is added to the existing learner. As (2) and (6) shown, the difference value added to the existing learner is formed from the difference of result and the random number *r*_*i*_. Therefore, in the teacher and learner phases, the difference value is decided by the random number *r*_*i*_ to a large extent. In our proposed method, we modify the random number *r*_*i*_ as follows:

(8) $$\begin{array}{c} r_{i}^{\prime }=\lambda +\left(1-\lambda \right)\mathrm{rand}\left(0,1\right), \end{array}$$

where rand⁡(0,1) is a uniformly distributed random number within the range [0, 1]. The value *λ* ∈ (0,1) should be neither too big nor too small. Here, *λ* is selected to be 0.5, which conforms to the dynamic inertia weight proposed by Eberhart and Shi [28]. So (8) is modified as

(9) $$\begin{array}{c} r_{i}^{\prime }=0.5+\frac{\mathrm{rand}\left(0,1\right)}{2}. \end{array}$$

Equation (9) generates a random number in the range [0.5, 1] which is similar to the method proposed by Satapathy and Naik [23]. We call *r*_*i*_′* dynamic inertia weighted factor*. Therefore, the mean value of the random number *r*_*i*_ is raised from 0.5 to 0.75. This increases the probability of stochastic variations and enlarges the difference value added to the existing learners, so as to improve population diversity, avoid prematurity in the search process, and increase the ability of the basic TLBO to escape from local optima. On the multimodal function surface, the original random weighed factor leads to most of the populations clustering near a local optimum point. However, the population with new dynamic inertia weight has more chances to jump out of the local optima and continuously move towards the global optimum point until a true global optimum is reached.

With the nonlinear inertia weighted factor and the dynamic inertia weighted factor, the new set of improved learners can be expressed by using equation in the teacher phase

(10) $$\begin{array}{c} X_{i,k,j}^{new}=wX_{i,k,j}^{old}+r_{i}^{\prime }\left(X_{i,teacher,j}-T_{F}M_{i,j}\right) \end{array}$$

and the new set of improved learners can be expressed by using equation in the learner phase

(11) $$\begin{array}{c} X_{i,k,j}^{new}=\left\{\begin{array}{cc} wX_{i,k,j}^{old}+r_{i}^{\prime }\left(X_{i,k,j}-X_{i,q,j}\right) & if\;\;f\left(X_{i,total\_k}\right)<f\left(X_{i,total\_q}\right),\\ wX_{i,k,j}^{old}+r_{i}^{\prime }\left(X_{i,q,j}-X_{i,k,j}\right) & otherwise, \end{array}\right. \end{array}$$

where *w* is given by (7) and *r*_*i*_′ is given by (9).

## 4. Experiments on Benchmark Functions

In this section, NIWTLBO is applied on several benchmark functions to evaluate its performance with different dimensions and search space, comparing with the basic TLBO algorithm and with other optimization algorithms available in the literature. All tests are evaluated on a laptop having Intel core i5 2.67 GHz processor and 2 GB RAM. The algorithm is coded using the MATLAB programming language and run in MATLAB 2012a environment. This section provides the results obtained by the NIWTLBO algorithm compared to the basic TLBO and other intelligent optimization algorithms. The details of the 24 benchmark functions with different characteristics like unimodality/multimodality and separability/nonseparability are shown in Table 1. “C” denotes the characteristic of function; “*D*” is the dimensions of function; “range” of each function is the difference between the lower and upper bounds of the variables; “*f*_min_” is the theoretical global minimum solution.

### 4.1. Experiment 1: NIWTLBO versus PSO, ABC, DE, and TLBO

This experiment is aimed at identifying the performance of the NIWTLBO algorithm to achieve the global optimum value comparing with PSO, ABC, DE, and the basic TLBO. To be fair, each algorithm uses the same values of common control parameters such as population size and maximum evaluation number. Population size is 40 and the maximum fitness function evaluation number is 80,000 for all benchmark functions in Table 1. The other specific parameters of algorithms are given below.

*PSO Setting*. Cognitive attraction *C*_1_ = 2, social attraction *C*_2_ = 2, and inertia weight *w* = 0.9. As mentioned in [5], a recommended choice for constant *C*_1_ and *C*_2_ is integer 2, since it on average makes the weights for “social” and “cognition” parts be 1. When *w* is in the range of [0.8,1.2], the PSO will have the best chance to find the global optimum and takes a moderate number of iterations [29].

*ABC Setting*. For ABC there are no other specific parameters to set.

*DE Setting*. In DE, *F* is a real constant which affects the differential variation between two solutions and *R* is crossover rate. Set *F* = 0.5 and *R* = 0.4. The configuration parameters for DE are decided on the results of experiments using different parameter values. We choose the parameter values which make the DE algorithms get the best result.

*TLBO Settings*. For TLBO there are no other specific parameters to set.

*NIWTLBO Settings*. In NIWTLBO, there are no other specific parameters too.

In this section, each benchmark function is independently experimented 30 times with PSO, ABC, DE, TLBO, and NIWTLBO. Each algorithm was terminated after running for 80,000FEs or when it reached the global minimum value before completely running for 80,000FEs. The mean and standard deviation of fitness value obtained through 30 experiments on each benchmark function are recorded in Table 2. Meanwhile, the mean value and standard deviations of the number of function fitness evaluations produced by the experiments are reported in Table 3. In order to analyze the performance whether there is significance between the results of the NIWTLBO and other algorithms, we carried out *t*-test on pairs of algorithms which is very popular in evolutionary computing [12]. The statistical significance levels of difference of the means of PSO and NIWTLBO algorithm, ABC and NIWTLBO algorithm, DE and NIWTLBO algorithm, and TLBO and NIWTLBO algorithm are reported in Table 4. Here, “+” symbol indicates that *t* value is significant at 0.05 level of significance by two tailed tests, “·” symbol marks *t* value being not statistically significant, and “NA” means not applicable due to the results of one pair of algorithms having the same accuracy.

The comparative results of each benchmark function for PSO, ABC, DE, and TLBO are presented in Table 2 in the form of average solution and standard deviation obtained in 30 independent runs on each benchmark function. The significance of NIWTLBO comparing with PSO, ABC, DE, and TLBO is shown in Table 4. It is observed from Tables 2 and 4 that the performance of NIWTLBO outperforms PSO, ABC, DE, and TLBO for functions *f*_1_–*f*_8_, *f*_18_, *f*_19_, *f*_21_, and *f*_23_. Furthermore, TLBO performs better than PSO, ABC, and DE for functions *f*_1_–*f*_8_ and *f*_18_. For functions *f*_10_–*f*_17_, the performance of NIWTLBO, PSO, ABC, DE, and TLBO is alike that almost all the algorithms can obtain the global optimum value except for ABC on Bohachevsky3. For Rosenbrock, the performance of different algorithms is similar to each other. For Griewank and Multimod, the performance of NIWTLBO, DE, and TLBO is alike and better than PSO and ABC. For Weierstrass, the performance of NIWTLBO and TLBO is alike and outperforms PSO, ABC, and DE.

It is observed from the results in Table 3 that the smaller the number of fitness evaluations the more quickly the algorithm obtains the global optimum value; that is, the convergence rate of the algorithm is faster. Obviously, the NIWTLBO algorithm requires less numbers of function evaluations than the basic TLBO algorithm and other algorithms mentioned to achieve the global optimum value for most of the benchmark functions. Hence, the convergence rate of the NIWTLBO algorithm is faster than other algorithms mentioned for most of the benchmark functions except Six-Hump Camel Back, Branin, and Goldstein-Price.

### 4.2. Experiment 2: NIWTLBO versus PSO-*w*, PSO-cf, CPSO-H, and CLPSO

In this section, the experiment is aimed at analysing the ability of the NIWTLBO algorithm to obtain the global optimum value comparing with other variant PSO algorithms such as PSO-*w* [29], PSO-cf [30], CPSO-H [31], and CLPSO [32]. In this experiment, 8 different unimodal and multimodal benchmark functions are tested using the NIWTLBO algorithm. The details of benchmark functions are shown in Table 1. In order to maintain the consistency in the comparison, NIWTLBO algorithm is performed with the same maximum function evaluations and dimensions. Each benchmark function is independently experimented 30 times for NIWTLBO. The comparative results are reported in Table 5 in the form of the average solution and standard deviation obtained in 30 independent runs on each benchmark function. In Table 5, the results of algorithms except NIWTLBO are taken from literatures [24, 27], where the algorithms run 30,000FEs with 10 population sizes for 10 dimensional functions.

It is observed from the results in Table 5 that the performance of NIWTLBO and TLBO algorithms is better than PSO-*w*, PSO-cf, CPSO-H, and CLPSO algorithms for Sphere, Ackley, and Griewank. The performance of NIWTLBO and CLPSO is alike for Rastrigin, Noncontinuous Rastrigin, and Weierstrass. For Rosenbrock and Schwefel 2.26, the NIWTLBO algorithm does not perform well comparing with other algorithms.

### 4.3. Experiment 3: NIWTLBO versus CABC, GABC, RABC, and IABC

In this section, the experiment is conducted to identify the performance of the NIWTLBO algorithm to achieve the global optimum value versus CABC [33], GABC [34], RABC [8], and IABC [35] on 7 benchmark functions shown in Table 1. The comparative results are reported in Table 6. To maintain the consistency in the comparison, the parameters of the algorithms are similar to the literature [8], where the population size is set as 20 and dimension is set as 30. The results of CABC, GABC, RABC, and IABC are taken from the literature [23] directly. The results of NIWTLBO and TLBO, in the form of average solution and standard deviation, are obtained in 30 independent runs on each benchmark function. In this experiment, TLBO and NIWTLBO are tested with the same function evaluations listed in Table 6 to compare their performance with CABC, GABC, RABC, and IABC algorithms.

From Table 6, it is observed obviously that the performance of NIWTLBO and TLBO algorithms is better than CABC, GABC, and RABC for all benchmark functions. The performance of NIWTLBO algorithm is similar to IABC for Rastrigin and Griewank and outperforms the IABC for the rest of benchmark functions in Table 6.

### 4.4. Experiment 4: NIWTLBO versus SaDE, jDE, and JADE

In this section, the experiment is carried out for comparing the performance of the NIWTLBO algorithm with SaDE, jDE, and JADE algorithms on 7 benchmark functions which are described in Table 1. The results of SaDE, jDE, and JADE are taken from the literature [36] directly. The results of NIWTLBO and TLBO, in the form of average solution and standard deviation, are obtained in 30 independent runs on each benchmark function. To be fair, the parameters of the algorithms are the same to the literature [36], where the population size is 20 and the dimension is 30. The comparative results are recorded in Table 7. In this experiment, TLBO and NIWTLBO are implemented with the same function evaluations listed in Table 7 to compare their performance with SaDE, jDE, and JADE algorithms.

It can be seen that NIWTLBO performs much better than these variants of DE on all the benchmark functions in Table 7. Therefore, it is shown that the NIWTLBO algorithm has a good performance.

### 4.5. Experiment 5: NIWTLBO versus TLBO with Different Dimensions

In this section, we analyse the convergence of NIWTLBO and TLBO algorithms with different dimensions. Two unimodal functions and two multimodal functions have been tested with dimensions 2, 10, 50, and 100. In this work, evolutionary generation is employed to evaluate the performance of NIWTLBO and TLBO algorithms. The population size is set as 40 and the number of evolutionary generations is set as 2000. The experiment results of NIWTLBO and TLBO algorithms for 2, 10, 50, and 100 dimensional functions over 30 independent runs are listed in Table 8, which is in form of the mean solution. The graphs are plotted between the function value and evolutionary generations on logarithmic scale.

**Figure 2:**
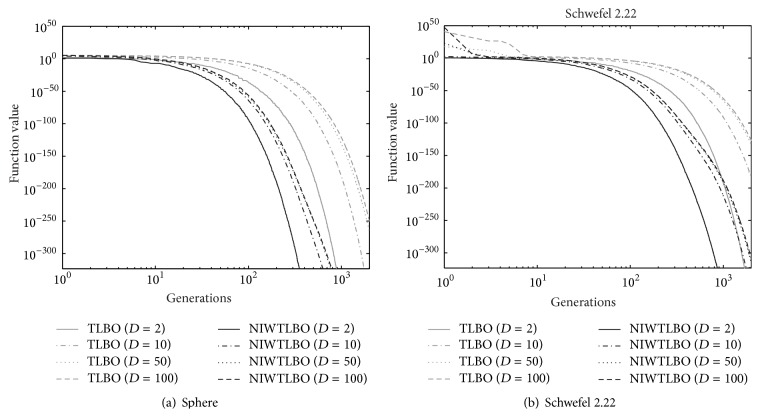
Convergence of TLBO and NIWTLBO algorithms for unimodal function.

**Figure 3:**
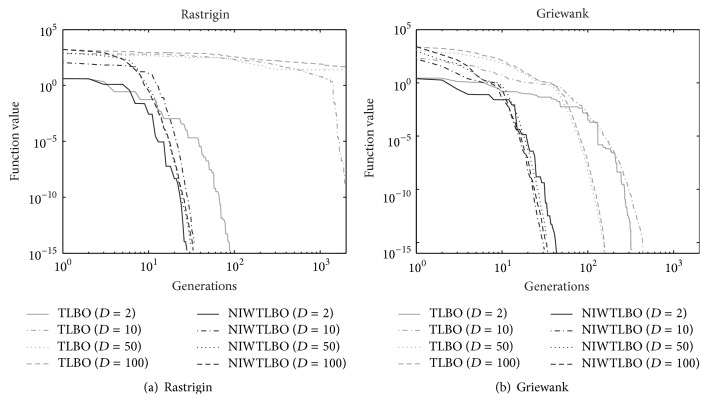
Convergence of TLBO and NIWTLBO algorithms for multimodal function.

Figures 2 and 3 show the convergence graphs of the unimodal and multimodal functions for different dimensions, respectively. It is observed from the graphs that the convergence rate of the NIWTLBO algorithm is faster than the basic TLBO algorithm for both these unimodal and multimodal functions for all dimensions. Furthermore, it is observed from Table 8 and the figures that the performance of NIWTLBO algorithm is almost not affected by the dimension. But the performance of TLBO algorithm will be reduced slightly with the dimension increasing.

### 4.6. Experiment 6: NIWTLBO versus Other Variants of TLBO

In order to show the advantages and disadvantages of the NIWTLBO, we make experiments to compare the performance of the NIWTLBO algorithm with some other variants of TLBO in this section. The variants of TLBO include WTLBO [21], ITLBO22 [22], ITLBO23 [23], and ITLBO [24]. Some benchmark functions described in Table 1 are tested for experiments. In the experiments, the population size is 20 and dimension is 2. The number of teachers is 4 in ITLBO. To maintain the consistency, the execution of the NIWTLBO and other variants of TLBO algorithms is stopped after running for 80,000FEs or when the difference between the fitness obtained by the algorithm and the global optimum value is less than 0.1% (e.g., if the optimum value is 0, the solution is accepted if it differs from the optimum value by less than 0.001). If the solution to the algorithm is not accepted after running for 80,000FEs, it is unsuccessful. Each benchmark function is tested 100 times with the NIWTLBO and other variants of TLBO algorithms and the comparative results in the form of mean function evaluations and success percentage are shown in Table 9. “MNFE” denotes the number of function evaluations when the solution is accepted. The number of function evaluations in the variants of TLBO is = (2 × population size × number of generations).

It is observed from Table 9 that, except for Rosenbrock and Branin, the NIWTLBO algorithm requires fewer number of function evaluations than other algorithms to reach the global optimum value, with a very high success rate of 100%. For Rosenbrock, Branin, Griewank, and Weierstrass, the WTLBO algorithm performs worse than other algorithms with low success rate, which is easily trapped in local optima. From this, it is shown that the NIWTLBO algorithm has a better performance than some other variants of TLBO.

## 5. Conclusion

In this paper, we propose the NIWTLBO algorithm which introduced a nonlinear inertia weighted factor into the basic TLBO to control the memory rate of learners and used a dynamic inertia weighted factor to replace the original random number in teacher phase and learner phase. The proposed algorithm is implemented on 24 benchmark functions having different characteristics to evaluate its performance which is compared with the basic TLBO and some other state-of-the-art optimization algorithms available in the literature. Furthermore, the comparisons between the NIWTLBO and other algorithms mentioned are also reported.

The experiment results have shown the satisfactory performance of the NIWTLBO algorithm for solving global optimization problems. The NIWTLBO algorithm not only enhances the local searching ability of TLBO but also improves the global performance. Moreover, the NIWTLBO algorithm can increase the convergence speed and enhance the ability of the TLBO to escape from local optima.

In future work, the NIWTLBO algorithm will be extended to handle more complex functions and solve constrained/multiobjective optimization problems. Furthermore, we will also open up a new way to improve the diversity of TLBO using a hybrid method, so as to utilize the advantages of other intelligent algorithms to further improve the global performance of TLBO.

## Figures and Tables

**Table 1:** List of benchmark functions which have been used in experiments.

Number	Function	C	*D*	Range	Formulation	*f*_min⁡_
*f*_1_	Sphere	US	30	[−100, 100]	*f*(*x*) = ∑_*i*=1_^*D*^ *x*_*i*_^2^	*f*_min⁡_ = 0

*f*_2_	SumSquares	US	30	[−100, 100]	*f*(*x*) = ∑_*i*=1_^*D*^ *ix*_*i*_^2^	*f*_min⁡_ = 0

*f*_3_	Tablet	US	30	[−100, 100]	*f*(*x*) = 10^6^ *x*_1_^2^ + ∑_*i*=1_^*D*^ *x* ^2^	*f*_min⁡_ = 0

*f*_4_	Quartic	US	30	[−1.28 1.28]	*f*(*x*) = ∑_*i*=1_^*D*^ *ix*_*i*_^4^ + random(0,1)	*f*_min⁡_ = 0

*f*_5_	Schwefel 1.2	UN	30	[−100, 100]	*f*(*x*) = ∑_*i*=1_^*D*^(∑_*j*=1_^*i*^ *x*_*j*_)^2^	*f*_min⁡_ = 0

*f*_6_	Schwefel 2.22	UN	30	[−10, 10]	$f\left(x\right)=\sum_{i=1}^{D}\left|x_{i}\right|+\prod_{i=1}^{D}\left|x_{i}\right|$	*f*_min⁡_ = 0

*f*_7_	Schwefel 2.21	UN	30	[−100, 100]	$f\left(x\right)=\;\;\overset{D}{\underset{i}{\max}}\left\{\left|x_{i}\right|\right\},\;1\leq i\leq D$	*f*_min⁡_ = 0

*f*_8_	Zakharov	UN	30	[−5, 10]	*f*(*x*) = ∑_*i*=1_^*D*^ *x*_*i*_^2^ + (∑_*i*=1_^*D*^0.5*ix*_*i*_)^2^ + (∑_*i*=1_^*D*^0.5*ix*_*i*_)^4^	*f*_min⁡_ = 0

*f*_9_	Rosenbrock	US	30	[−4, 4]	*f*(*x*) = ∑_*i*=1_^*D*−1^[100(*x*_*i*+1_ − *x*_*i*_^2^)^2^ + (1 − *x*_*i*_)^2^]	*f*_min⁡_ = 0

*f*_10_	Schaffer	MN	2	[−10, 10]	$f\left(x\right)=\frac{\sin^{2}\left(\sqrt{x_{1}^{2}+x_{2}^{2}}\right)-0.5}{{\left(1+0.001\left(x_{1}^{2}+x_{2}^{2}\right)\right)}^{2}}-0.5$	*f*_min⁡_ = −1

*f*_11_	Dropwave	MN	2	[−2, 2]	$f\left(x\right)=-\frac{1+cos\left(12\sqrt{x_{1}^{2}+x_{2}^{2}}\right)}{0.5\left(x_{1}^{2}+x_{2}^{2}\right)+2}$	*f*_min⁡_ = −1

*f*_12_	Bohachevsky1	MN	2	[−100, 100]	*f*(*x*) = *x*_1_^2^ + 2*x*_2_^2^ − 0.3cos⁡(3*πx*_1_) − 0.4cos⁡(4*πx*_2_) + 0.7	*f*_min⁡_ = 0

*f*_13_	Bohachevsky2	MN	2	[−100, 100]	*f*(*x*) = *x*_1_^2^ + 2*x*_2_^2^ − 0.3cos⁡(3*πx*_1_)*∗*cos⁡(4*πx*_2_) + 0.3	*f*_min⁡_ = 0

*f*_14_	Bohachevsky3	MN	2	[−100, 100]	*f*(*x*) = *x*_1_^2^ + 2*x*_2_^2^ − 0.3cos⁡(3*πx*_1_ + 4*πx*_2_) + 0.3	*f*_min⁡_ = 0

*f*_15_	Six-Hump Camel Back	MN	2	[−5, 5]	$f\left(x\right)=4x_{1}^{2}-2.1x_{1}^{4}-\frac{1}{3}x_{1}^{6}+x_{1}x_{2}-4x_{2}^{2}+4x_{2}^{4}$	*f*_min⁡_ = −1.03163

*f*_16_	Branin	MS	2	[−5, 15]	$f\left(x\right)={\left(x_{2}-\frac{5.1}{4\pi^{2}}x_{1}^{2}+\frac{5}{\pi }x_{1}-6\right)}^{2}+10\left(1-\frac{1}{8\pi }\right)\cos x_{1}+10$	*f*_min⁡_ = 0.398

*f*_17_	Goldstein-Price	MN	2	[−2, 2]	*f*(*x*) = [1 + (*x*_1_ + *x*_2_ + 1)^2^(19 − 14*x*_1_ + 3*x*_1_^2^ − 14*x*_2_ + 6*x*_1_ *x*_2_ + 3*x*_2_^2^)] ×[30 + (2*x*_1_ − 3*x*_2_)^2^ × (18 − 32*x*_1_ + 12*x*_1_^2^ + 48*x*_2_ − 36*x*_1_ *x*_2_ + 27*x*_2_^2^)]	*f*_min⁡_ = 3

*f*_18_	Ackley	MN	30	[−32, 32]	$f\left(x\right)=-20\exp\left(-0.2\sqrt{\frac{1}{D}\sum_{i=1}^{D}x_{i}^{2}}\right)-\exp\left(\frac{1}{D}\sum_{i=1}^{D}\cos\left(2\pi x_{i}\right)\right)+20+e$	*f*_min⁡_ = 0

*f*_19_	Rastrigin	MN	30	[−5.12, 5.12]	*f*(*x*) = ∑_*i*=1_^*D*^(*x*_*i*_^2^ − 10cos⁡(2*πx*_*i*_) + 10)	*f*_min⁡_ = 0

*f*_20_	Griewank	MN	30	[−600, 600]	$f\left(x\right)=\frac{1}{4000}\sum_{i=1}^{D}x_{i}^{2}-\prod_{i=1}^{D}\cos\left(\frac{x_{i}}{\sqrt{i}}\right)+1$	*f*_min⁡_ = 0

*f*_21_	Schwefel 2.26	MN	30	[−500, 500]	$f\left(x\right)=-\sum_{i=1}^{D}\left(x_{i}\sin\left(\sqrt{\left|x_{i}\right|}\right)\right)$	*f*_min⁡_ = −837.9658

*f*_22_	Multimod	MN	30	[−10, 10]	$f\left(x\right)=\sum_{i=1}^{D}\left|x_{i}\right|\prod_{i=1}^{D}\left|x_{i}\right|$	*f*_min⁡_ = 0

*f*_23_	Noncontinuous Rastrigin	MS	30	[−5.12, 5.12]	*f*(*x*) = ∑_*i*=1_^*D*^(*y*_*i*_^2^ − 10cos⁡(2*πy*_*i*_) + 10), $\text{where}\;\;y_{i}=\left\{\begin{array}{cc} x_{i} & |x_{i}|<0.5\\ 0.5\text{round}\left(2x_{i}\right) & \left|x_{i}\right|\geq 0.5 \end{array}\right.$	*f*_min⁡_ = 0

*f*_24_	Weierstrass	MS	30	[−0.5, 0.5]	*f*(*x*) = ∑_*i*=1_^*D*^(∑_*k*=0_^*km*^[*a* ^*k*^cos⁡(2*πb* ^*k*^(*x*_*i*_ + 0.5))]) − *D*∑_*k*=0_^*km*^[*a* ^*k*^cos⁡(2*πb* ^*k*^0.5)] where *a* = 0.5, *b* = 3, *km* = 20	*f*_min⁡_ = 0

C: characteristic; *D*: dimension; U: unimodal; M: multimodal; S: separable; N: nonseparable.

**Table 2:** Performance comparisons of PSO, ABC, DE, TLBO, and NIWTLBO in terms of fitness value. Population size: 40; *D*: 30 (except *f*_10_~*f*_17_: 2*D*); max. eval.: 80,000FEs.

Number	Function	*f*_min⁡_		PSO	ABC	DE	TLBO	NIWTLBO
*f*_1_	Sphere	0	Mean	8.99*E* − 12	9.91*E* − 16	7.15*E* − 27	1.85*E* − 286	0
			Std.	5.92*E* − 12	5.36*E* − 16	1.06*E* − 26	0	0

*f*_2_	SumSquares	0	Mean	1.11*E* − 10	7.81*E* − 16	9.06*E* − 26	1.57*E* − 286	0
			Std.	2.49*E* − 10	1.32*E* − 16	3.07*E* − 26	0	0

*f*_3_	Tablet	0	Mean	3.68*E* − 08	9.54*E* − 16	2.40*E* − 26	7.66*E* − 285	0
			Std.	1.64*E* − 08	1.78*E* − 16	1.87*E* − 26	0	0

*f*_4_	Quartic	0	Mean	5.84*E* − 02	1.52*E* − 01	4.03*E* − 01	2.07*E* − 02	2.03*E* − 02
			Std.	3.83*E* − 02	4.18*E* − 02	1.29*E* − 01	5.26*E* − 02	3.52*E* − 02

*f*_5_	Schwefel 1.2	0	Mean	2.47*E* + 05	8.82*E* + 03	2.21*E* + 04	1.52*E* − 84	0
			Std.	1.48*E* + 05	1.28*E* + 03	5.21*E* + 03	2.97*E* − 84	0

*f*_6_	Schwefel 2.22	0	Mean	5.16*E* − 03	2.01*E* − 14	4.31*E* − 16	1.79*E* − 143	4.45*E* – 323
			Std.	6.94*E* − 03	1.08*E* − 14	1.04*E* − 16	1.21*E* − 143	0

*f*_7_	Schwefel 2.21	0	Mean	1.21*E* + 00	5.49*E* + 01	1.21*E* − 02	8.31*E* − 120	2.40*E* − 315
			Std.	6.02*E* − 01	1.38*E* + 01	2.81*E* − 03	4.05*E* − 120	0

*f*_8_	Zakharov	0	Mean	1.62*E* + 02	2.59*E* + 02	5.84*E* + 01	5.95*E* − 51	1.06*E* − 319
			Std.	6.33*E* + 01	2.84*E* + 01	7.01*E* + 00	5.22*E* − 51	0

*f*_9_	Rosenbrock	0	Mean	3.01*E* + 01	1.04*E* + 01	2.43*E* + 01	1.29*E* + 01	1.83*E* + 01
			Std.	2.57*E* + 01	2.57*E* + 00	4.61*E* + 00	5.28*E* + 00	6.91*E* + 00

*f*_10_	Schaffer	−1	Mean	−1	−1	−1	−1	−1
			Std.	0	0	0	0	0

*f*_11_	Dropwave	−1	Mean	−1	−1	−1	−1	−1
			Std.	0	0	0	0	0

*f*_12_	Bohachevsky1	0	Mean	0	0	0	0	0
			Std.	0	0	0	0	0

*f*_13_	Bohachevsky2	0	Mean	0	0	0	0	0
			Std.	0	0	0	0	0

*f*_14_	Bohachevsky3	0	Mean	0	8.46*E* − 16	0	0	0
			Std.	0	2.95*E* − 16	0	0	0

*f*_15_	Six-Hump Camel Back	−1.03163	Mean	−1.03163	−1.03163	−1.03163	−1.03163	−1.03163
			Std.	0	0	0	0	0

*f*_16_	Branin	0.398	Mean	0.3979	0.3979	0.3979	0.3979	0.3979
			Std.	0	0	0	0	0

*f*_17_	Goldstein-Price	3	Mean	3	3	3	3	3
			Std.	8.11*E* − 15	4.32*E* − 15	1.36*E* − 15	6.78*E* − 16	6.56*E* − 16

*f*_18_	Ackley	0	Mean	1.18*E* + 00	2.82*E* − 13	2.49*E* − 14	4.44*E* − 15	8.66*E* − 16
			Std.	3.85*E* − 01	3.06*E* − 14	6.07*E* − 15	0	0

*f*_19_	Rastrigin	0	Mean	1.08*E* + 02	1.29*E* − 13	9.33*E* + 01	6.93*E* + 00	0
			Std.	2.80*E* + 01	2.57*E* − 13	9.43*E* + 00	5.92*E* + 00	0

*f*_20_	Griewank	0	Mean	6.77*E* − 03	7.10*E* − 03	0	0	0
			Std.	9.29*E* − 03	9.56*E* − 03	0	0	0

*f*_21_	Schwefel 2.26	−837.9658	Mean	−8789.43	−12561.79	−11312.51	−9178.59	−8324.302
			Std.	4.63*E* + 02	1.96*E* + 02	1.58*E* + 03	7.97*E* + 02	1.71*E* + 02

*f*_22_	Multimod	0	Mean	8.69*E* − 67	8.52*E* − 19	4.66*E* − 311	0	0
			Std.	1.74*E* − 66	8.34*E* − 19	0	0	0

*f*_23_	Noncontinuous Rastrigin	0	Mean	1.83*E* + 02	1.99*E* − 14	6.94*E* + 01	1.55*E* + 01	0
			Std.	3.15*E* + 01	1.83*E* − 14	9.13*E* + 00	2.65*E* + 00	0

*f*_24_	Weierstrass	0	Mean	6.27*E* + 01	1.12*E* − 02	1.38*E* + 01	0	0
			Std.	2.03*E* + 01	7.73*E* − 03	6.07*E* − 01	0	0

**Table 3:** Convergence comparisons in terms of number of fitness evaluations. Population size: 40; *D*: 30 (except *f*_10_~*f*_17_: 2*D*); max. eval.: 80,000FEs.

Number	Function		PSO	ABC	DE	TLBO	NIWTLBO
*f*_1_	Sphere	Mean	80,000	80,000	80,000	80,000	29,514
		Std.	0	0	0	0	1.02*E* + 02

*f*_2_	SumSquares	Mean	80,000	80,000	80,000	80,000	29,628
		Std.	0	0	0	0	1.23*E* + 02

*f*_3_	Tablet	Mean	80,000	80,000	80,000	80,000	29,562
		Std.	0	0	0	0	1.52*E* + 02

*f*_4_	Quartic	Mean	80,000	80,000	80,000	80,000	80,000
		Std.	0	0	0	0	0

*f*_5_	Schwefel 1.2	Mean	80,000	80,000	80,000	80,000	39,416
		Std.	0	0	0	0	1.09*E* + 02

*f*_6_	Schwefel 2.22	Mean	80,000	80,000	80,000	80,000	80,000
		Std.	0	0	0	0	0

*f*_7_	Schwefel 2.21	Mean	80,000	80,000	80,000	80,000	80,000
		Std.	0	0	0	0	0

*f*_8_	Zakharov	Mean	80,000	80,000	80,000	80,000	80,000
		Std.	0	0	0	0	0

*f*_9_	Rosenbrock	Mean	80,000	80,000	80,000	80,000	80,000
		Std.	0	0	0	0	0

*f*_10_	Schaffer	Mean	12,432	43,636	8,686	9,688	3,029
		Std.	3.38*E* + 02	3.03*E* + 02	2.06*E* + 02	2.29*E* + 02	3.03*E* + 02

*f*_11_	Dropwave	Mean	11,394	13,824	5,490	3,021	812
		Std.	3.26*E* + 01	1.09*E* + 02	1.53*E* + 02	1.22*E* + 02	3.32*E* + 01

*f*_12_	Bohachevsky1	Mean	9,532	3,263	3,992	2,266	842
		Std.	2.21*E* + 02	7.52*E* + 01	8.74*E* + 01	3.23*E* + 01	2.01*E* + 01

*f*_13_	Bohachevsky2	Mean	9,578	4,717	4,245	2,568	952
		Std.	1.33*E* + 02	9.27*E* + 01	1.17*E* + 02	2.05*E* + 01	2.56*E* + 01

*f*_14_	Bohachevsky3	Mean	9,792	80,000	5,376	2,875	965
		Std.	2.52*E* + 02	0	1.26*E* + 02	1.03*E* + 02	3.12*E* + 01

*f*_15_	Six-Hump	Mean	1,997	1,372	1,781	712	2,560
	Camel Back	Std.	1.38*E* + 02	1.17*E* + 02	1.36*E* + 02	5.93*E* + 01	9.07*E* + 01

*f*_16_	Branin	Mean	1,851	1,813	1,891	1,086	2,172
		Std.	1.17*E* + 02	1.23*E* + 02	1.04*E* + 02	1.06*E* + 02	1.23*E* + 02

*f*_17_	Goldstein-Price	Mean	2,018	1,857	1,765	1,228	2,865
		Std.	1.25*E* + 02	1.48*E* + 02	2.08*E* + 02	6.85*E* + 01	1.42*E* + 02

*f*_18_	Ackley	Mean	80,000	80,000	80,000	80,000	80,000
		Std.	0	0	0	0	0

*f*_19_	Rastrigin	Mean	80,000	80,000	80,000	80,000	1,436
		Std.	0	0	0	0	3.02*E* + 01

*f*_20_	Griewank	Mean	80,000	80,000	53,032	12,064	1,284
		Std.	0	0	6.16*E* + 02	9.37*E* + 01	2.54*E* + 01

*f*_21_	Schwefel 2.26	Mean	80,000	80,000	80,000	80,000	80,000
		Std.	0	0	0	0	0

*f*_22_	Multimod	Mean	80,000	80,000	80,000	28,304	1,427
		Std.	0	0	0	1.05*E* + 02	5.16*E* + 01

*f*_23_	Noncontinuous Rastrigin	Mean	80,000	80,000	80,000	80,000	1,324
		Std.	0	0	0	0	1.22*E* + 02

*f*_24_	Weierstrass	Mean	80,000	80,000	80,000	12,712	2,044
		Std.	0	0	0	1.19*E* + 02	1.21*E* + 02

**Table 4:** *t* value, significant at 0.05 level of significance by two tailed tests using Table 2. The significance of NIWTLBO compares with PSO, ABC, DE, and TLBO.

Number	Function	PSO	ABC	DE	TLBO	Number	Function	PSO	ABC	DE	TLBO
*f*_1_	Sphere	+	+	+	+	*f*_13_	Bohachevsky2	NA	NA	NA	NA
*f*_2_	SumSquares	+	+	+	+	*f*_14_	Bohachevsky3	NA	+	NA	NA
*f*_3_	Tablet	+	+	+	+	*f*_15_	Six-Hump Camel Back	NA	NA	NA	NA
*f*_4_	Quartic	+	+	+	·	*f*_16_	Branin	NA	NA	NA	NA
*f*_5_	Schwefel 1.2	+	+	+	+	*f*_17_	Goldstein-Price	NA	NA	NA	NA
*f*_6_	Schwefel 2.22	+	+	+	+	*f*_18_	Ackley	+	+	+	+
*f*_7_	Schwefel 2.21	+	+	+	+	*f*_19_	Rastrigin	+	+	+	+
*f*_8_	Zakharov	+	+	+	+	*f*_20_	Griewank	+	+	NA	NA
*f*_9_	Rosenbrock	·	·	·	·	*f*_21_	Schwefel 2.26	+	+	+	+
*f*_10_	Schaffer	NA	NA	NA	NA	*f*_22_	Multimod	+	+	NA	NA
*f*_11_	Dropwave	NA	NA	NA	NA	*f*_23_	Noncontinuous Rastrigin	+	+	+	+
*f*_12_	Bohachevsky1	NA	NA	NA	NA	*f*_24_	Weierstrass	+	+	+	NA

“+” indicates that *t* value is significant, “·” indicates that *t* value is not statistically significant, and “NA” stands for not applicable.

**Table 5:** Comparative results of TLBO and NIWTLBO with other PSO algorithms. Population size: 10; *D*: 10; max. eval.: 30,000FEs; source: results of algorithms except NIWTLBO are taken from [24, 27].

Number	Function		PSO-w	PSO-cf	CPSO-H	CLPSO	TLBO	NIWTLBO
*f*_1_	Sphere	Mean	7.96*E* − 51^†^	9.84*E* − 105^†^	4.98*E* − 45^†^	5.15*E* − 29^†^	0	0
		Std.	3.56*E* − 50	4.21*E* − 104	1.00*E* − 44	2.16*E* − 28	0	0

*f*_9_	Rosenbrock	Mean	3.08*E* + 00^†^	6.98*E* − 01^‡^	1.53*E* + 00^‡^	2.46*E* + 00^†^	1.72*E* + 00^‡^	1.69*E* + 00
		Std.	7.69*E* − 01	1.46*E* + 00	1.70*E* + 00	1.70*E* + 00	6.62*E* − 01	7.18*E* − 01

*f*_18_	Ackley	Mean	1.58*E* − 14^†^	9.18*E* − 01^†^	1.49*E* − 14^†^	4.32*E* − 10^†^	3.55*E* − 15^†^	8.58*E* − 16
		Std.	1.60*E* − 14	1.01*E* + 00	6.97*E* − 15	2.55*E* − 14	8.32*E* − 31	6.37*E* − 32

*f*_19_	Rastrigin	Mean	5.82*E* + 00^†^	1.25*E* + 01^†^	2.12*E* + 00^†^	0	6.77*E* − 08^†^	0
		Std.	2.96*E* + 00	5.17*E* + 00	1.33*E* + 00	0	3.68*E* − 07	0

*f*_20_	Griewank	Mean	9.69*E* − 02^†^	1.19*E* − 01^†^	4.07*E* − 02^†^	4.56*E* − 03^†^	0	0
		Std.	5.01*E* − 02	7.11*E* − 02	2.80*E* − 02	4.81*E* − 03^†^	0	0

*f*_21_	Schwefel 2.26	Mean	3.20*E* + 02^†^	9.87*E* + 02^†^	2.13*E* + 02^‡^	0^‡^	2.94*E* + 02^†^	2.67*E* + 02
		Std.	1.85*E* + 02	2.76*E* + 02	1.41*E* + 02	0	2.68*E* + 02	1.92*E* + 02

*f*_23_	Noncontinuous Rastrigin	Mean	4.05*E* + 00^†^	1.20*E* + 01^†^	2.00*E* − 01^†^	0	2.65*E* − 08^†^	0
		Std.	2.58*E* + 00	4.99*E* + 00	4.10*E* − 01	0	1.23*E* − 07	0

*f*_24_	Weierstrass	Mean	2.28*E* − 03^†^	6.69*E* − 01^†^	1.07*E* − 15^†^	0	2.42*E* − 05^†^	0
		Std.	7.04*E* − 03	7.17*E* − 01	1.67*E* − 15	0	1.38*E* − 20	0

“†” mark indicates that NIWTLBO is statistically better than the corresponding algorithm.

“‡” mark indicates that NIWTLBO is statistically worse than the corresponding algorithm.

**Table 6:** Comparative results of TLBO and NIWTLBO with other variants of ABC algorithms. Population size: 20; *D*: 30; source: results of algorithms except TLBO and NIWTLBO are taken from [23].

Number	Function		CABC	GABC	RABC	IABC	TLBO	NIWTLBO
*f*_1_	Sphere	Mean	2.3*E* − 40^†^	3.6*E* − 63^†^	9.1*E* − 61^†^	5.34*E* − 178^†^	0	0
	FEs: 1.5 × 10^5^	Std.	1.7*E* − 40	5.7*E* − 63	2.1*E* − 60	0	0	0

*f*_5_	Schwefel 1.2	Mean	8.4*E* + 02^†^	4.3*E* + 02^†^	2.9*E* − 24^†^	1.78*E* − 65^†^	0	0
	FEs: 5.0 × 10^5^	Std.	9.1*E* + 02	8.0*E* + 02	1.5*E* − 23	2.21*E* − 65	0	0

*f*_6_	Schwefel 2.22	Mean	3.5*E* − 30^†^	4.8*E* − 45^†^	3.2*E* − 74^†^	8.82*E* − 127^†^	0	0
	FEs: 2.0 × 10^5^	Std.	4.8*E* − 30	1.4*E* − 45	2.0*E* − 73	3.49*E* − 126	0	0

*f*_7_	Schwefel 2.21	Mean	6.1*E* − 03^†^	3.6*E* − 06^†^	2.8*E* − 02^†^	4.98*E* − 38^†^	0	0
	FEs: 5.0 × 10^5^	Std.	5.7*E* − 03	7.6*E* − 07	1.7*E* − 02	8.59*E* − 38	0	0

*f*_18_	Ackley	Mean	1.0*E* − 05^†^	1.8*E* − 09^†^	9.6*E* − 07^†^	3.87*E* − 14^†^	4.48*E* − 15^†^	8.65*E* − 16
	FEs: 5.0 × 10^4^	Std.	2.4*E* − 06	7.7*E* − 10	8.3*E* − 07	8.52*E* − 15	2.16*E* − 30	2.38*E* − 31

*f*_19_	Rastrigin	Mean	1.3*E* − 00^†^	1.5*E* − 10^†^	2.3*E* − 02^†^	0	6.36*E* + 00^†^	0
	FEs: 1.0 × 10^5^	Std.	2.7*E* − 00	2.7*E* − 10	5.1*E* − 01	0	4.78*E* + 00	0

*f*_20_	Griewank	Mean	1.2*E* − 04^†^	6.0*E* − 13^†^	8.7*E* − 08^†^	0	0	0
	FEs: 5.0 × 10^5^	Std.	4.6*E* − 04	7.7*E* − 13	2.1*E* − 08	0	0	0

“†” mark indicates that NIWTLBO is statistically better than the corresponding algorithm.

**Table 7:** Comparative results of TLBO and NIWTLBO with other variants of DE algorithms. Population size: 20; *D*: 30; source: results of algorithms except TLBO and NIWTLBO are taken from [23].

Number	Function		SaDE	jDE	JADE	TLBO	NIWTLBO
*f*_1_	Sphere	Mean	4.5*E* − 20^†^	2.5*E* − 28^†^	1.8*E* − 60^†^	0	0
	FEs: 1.5 × 10^5^	Std.	1.9*E* − 14	3.5*E* − 28	8.4*E* − 60	0	0

*f*_5_	Schwefel 1.2	Mean	9.0*E* − 37^†^	5.2*E* − 14^†^	5.7*E* − 61^†^	0	0
	FEs: 5.0 × 10^5^	Std.	5.4*E* − 36	1.1*E* − 13	2.7*E* − 60	0	0

*f*_6_	Schwefel 2.22	Mean	1.9*E* − 14^†^	1.5*E* − 23^†^	1.8*E* − 25^†^	0	0
	FEs: 2.0 × 10^5^	Std.	1.1*E* − 14	1.0*E* − 23	8.8*E* − 25	0	0

*f*_7_	Schwefel 2.21	Mean	7.4*E* − 11^†^	1.4*E* − 15^†^	8.2*E* − 24^†^	0	0
	FEs: 5.0 × 10^5^	Std.	1.82*E* − 10	1.0*E* − 15	4.0*E* − 23	0	0

*f*_18_	Ackley	Mean	2.7*E* − 03^†^	3.5*E* − 04^†^	8.2*E* − 10^†^	4.48*E* − 15^†^	8.65*E* − 16
	FEs: 5.0 × 10^4^	Std.	5.1*E* − 04	1.0*E* − 04	6.9*E* − 10	2.16*E* − 30	2.38*E* − 31

*f*_19_	Rastrigin	Mean	1.2*E* − 03^†^	1.5*E* − 04^†^	1.0*E* − 04^†^	6.36*E* + 00^†^	0
	FEs: 1.0 × 10^5^	Std.	6.5*E* − 04	2.0*E* − 04	6.0*E* − 05	4.78*E* + 00	0

*f*_20_	Griewank	Mean	7.8*E* − 04^†^	1.9*E* − 05^†^	9.9*E* − 08^†^	0	0
	FEs: 5.0 × 10^5^	Std.	1.2*E* − 03	5.8*E* − 05	6.0*E* − 07	0	0

“†” mark indicates that NIWTLBO is statistically better than the corresponding algorithm.

**Table 8:** Comparative results of TLBO and NIWTLBO with different dimensions. Population size: 40; generations: 2000.

Function	*D*	Unimodal		Multimodal	
		Sphere	Schwefel 2.22	Rastrigin	Griewank
TLBO	2	0	0	0	0
	10	0	1.05*E* − 184	5.78*E* − 08	0
	50	2.09*E* − 267	4.64*E* − 134	2.48*E* + 01	0
	100	4.13*E* − 251	8.91*E* − 128	4.71*E* + 01	0

NIWTLBO	2	0	0	0	0
	10	0	2.50*E* − 323	0	0
	50	0	4.43*E* − 317	0	0
	100	0	4.09*E* − 310	0	0

**Table 9:** Comparative results of NIWTLBO and different variants of TLBO algorithms. Population size: 20; *D*: 2; max. eval.: 80,000FEs.

Number	Function		WTLBO	ITLBO22	ITLBO23	I-TLBO (NT = 4)	NIWTLBO
*f*_1_	Sphere	MNFE	365	386	482	372	281
		Succ%	100	100	100	100	100

*f*_6_	Schwefel 2.22	MNFE	442	428	563	416	324
		Succ%	100	100	100	100	100

*f*_9_	Rosenbrock	MNFE	1643	704	726	684	1606
		Succ%	65	100	100	100	100

*f*_14_	Bohachevsky3	MNFE	468	432	516	398	364
		Succ%	100	100	100	100	100

*f*_16_	Branin	MNFE	41010	649	763	367	1922
		Succ%	28	100	100	100	100

*f*_18_	Ackley	MNFE	564	508	682	491	443
		Succ%	100	100	100	100	100

*f*_19_	Rastrigin	MNFE	4608	651	1406	632	481
		Succ%	100	100	100	100	100

*f*_20_	Griewank	MNFE	18246	1208	2248	1024	965
		Succ%	85	100	81	100	100

*f*_24_	Weierstrass	MNFE	19642	1243	2325	1186	1042
		Succ%	78	100	93	100	100

## References

[1] Blum C. (2005). Ant colony optimization: introduction and recent trends. *Physics of Life Reviews*.

[2] Mohan B. C., Baskaran R. (2012). A survey: ant colony optimization based recent research and implementation on several engineering domain. *Expert Systems with Applications*.

[3] Goswami B., Mandal D. (2013). A genetic algorithm for the level control of nulls and side lobes in linear antenna arrays. *Journal of King Saud University—Computer and Information Sciences*.

[4] Thakur M. (2014). A new genetic algorithm for global optimization of multimodal continuous functions. *Journal of Computational Science*.

[5] Kennedy J., Eberhart R. Particle swarm optimization.

[6] Beheshti Z., Shamsuddin S. M. (2015). Non-parametric particle swarm optimization for global optimization. *Applied Soft Computing Journal*.

[7] Tanweer M., Suresh S., Sundararajan N. (2015). Self regulating particle swarm optimization algorithm. *Information Sciences*.

[8] Kang F., Li J., Ma Z. (2011). Rosenbrock artificial bee colony algorithm for accurate global optimization of numerical functions. *Information Sciences*.

[9] Akay B., Karaboga D. (2012). Artificial bee colony algorithm for large-scale problems and engineering design optimization. *Journal of Intelligent Manufacturing*.

[10] Karaboga D., Gorkemli B., Ozturk C., Karaboga N. (2014). A comprehensive survey: artificial bee colony (ABC) algorithm and applications. *Artificial Intelligence Review*.

[11] Storn R., Price K. (1997). Differential evolution—a simple and efficient heuristic for global optimization over continuous spaces. *Journal of Global Optimization*.

[12] Das S., Abraham A., Chakraborty U. K., Konar A. (2009). Differential evolution using a neighborhood-based mutation operator. *IEEE Transactions on Evolutionary Computation*.

[13] Rao R. V., Savsani V. J., Vakharia D. P. (2011). Teaching–learning-based optimization: a novel method for constrained mechanical design optimization problems. *Computer-Aided Design*.

[14] Rao R. V., Savsani V. J., Vakharia D. P. (2012). Teaching-learning-based optimization: an optimization method for continuous non-linear large scale problems. *Information Sciences*.

[15] Rao R. V., Patel V. (2012). An elitist teaching-learning-based optimization algorithm for solving complex constrained optimization problems. *International Journal of Industrial Engineering Computations*.

[16] Venkata Rao R., Kalyankar V. D. (2013). Parameter optimization of modern machining processes using teaching—learning-based optimization algorithm. *Engineering Applications of Artificial Intelligence*.

[17] Rao R. V., Patel V. (2013). Multi-objective optimization of heat exchangers using a modified teaching-learning-based optimization algorithm. *Applied Mathematical Modelling*.

[18] Shabanpour-Haghighi A., Seifi A. R., Niknam T. (2014). A modified teaching-learning based optimization for multi-objective optimal power flow problem. *Energy Conversion and Management*.

[19] Sultana S., Roy P. K. (2014). Multi-objective quasi-oppositional teaching learning based optimization for optimal location of distributed generator in radial distribution systems. *International Journal of Electrical Power & Energy Systems*.

[20] Ghasemi M., Ghavidel S., Gitizadeh M., Akbari E. (2015). An improved teaching-learning-based optimization algorithm using Lévy mutation strategy for non-smooth optimal power flow. *International Journal of Electrical Power & Energy Systems*.

[21] Satapathy S. C., Naik A., Parvathi K. (2013). Weighted teaching-learning-based optimization for global function optimization. *Applied Mathematics*.

[22] Chen D., Zou F., Li Z., Wang J., Li S. (2015). An improved teaching–learning-based optimization algorithm for solving global optimization problem. *Information Sciences*.

[23] Satapathy S. C., Naik A. (2013). Improved teaching learning based optimization for global function optimization. *Decision Science Letters*.

[24] Rao R. V., Patel V. (2013). An improved teaching-learning-based optimization algorithm for solving unconstrained optimization problems. *Scientia Iranica*.

[25] Zou F., Wang L., Hei X., Chen D., Wang B. (2013). Multi-objective optimization using teaching-learning-based optimization algorithm. *Engineering Applications of Artificial Intelligence*.

[26] Rao R. V., Patel V. (2014). A multi-objective improved teaching-learning based optimization algorithm for unconstrained and constrained optimization problems. *International Journal of Industrial Engineering Computations*.

[27] Akay B., Karaboga D. (2012). A modified Artificial Bee Colony algorithm for real-parameter optimization. *Information Sciences*.

[28] Eberhart R. C., Shi Y. Tracking and optimizing dynamic systems with particle swarms.

[29] Shi Y., Eberhart R. C. A modified particle swarm optimizer.

[30] Clerc M., Kennedy J. (2002). The particle swarm-explosion, stability, and convergence in a multidimensional complex space. *IEEE Transactions on Evolutionary Computation*.

[31] Frans V. D. B., Engelbrecht A. P. (2004). A cooperative approach to particle swarm optimization. *IEEE Transactions on Evolutionary Computation*.

[32] Liang J. J., Suganthan P. N., Qin A. K., Baskar S. (2006). Comprehensive learning particle swarm optimizer for global optimization of multimodal functions. *IEEE Transactions on Evolutionary Computation*.

[33] Alatas B. (2010). Chaotic bee colony algorithms for global numerical optimization. *Expert Systems with Applications*.

[34] Zhu G. P., Kwong S. (2010). Gbest-guided artificial bee colony algorithm for numerical function. *Applied Soft Computing*.

[35] Gao W., Liu S. (2011). Improved artificial bee colony algorithm for global optimization. *Information Processing Letters*.

[36] Zhan Z.-H., Zhang J., Li Y., Chung H. S.-H. (2009). Adaptive particle swarm optimization. *IEEE Transactions on Systems, Man, and Cybernetics, Part B: Cybernetics*.

